# Golgi Apparatus: A Potential Therapeutic Target for Autophagy-Associated Neurological Diseases

**DOI:** 10.3389/fcell.2020.564975

**Published:** 2020-09-09

**Authors:** Shuwen Deng, Jia Liu, Xiaomei Wu, Wei Lu

**Affiliations:** Department of Neurology, The Second Xiangya Hospital, Central South University, Changsha, China

**Keywords:** autophagy, cellular processes, golgi, neurological diseases, therapy

## Abstract

Autophagy has dual effects in human diseases: appropriate autophagy may protect cells from stress, while excessive autophagy may cause cell death. Additionally, close interactions exist between autophagy and the Golgi. This review outlines recent advances regarding the role of the Golgi apparatus in autophagy. The signaling processes of autophagy are dependent on the normal function of the Golgi. Specifically, (i) autophagy-related protein 9 is mainly located in the Golgi and forms new autophagosomes in response to stressors; (ii) Golgi fragmentation is induced by Golgi-related proteins and accompanied with autophagy induction; and (iii) the endoplasmic reticulum-Golgi intermediate compartment and the reticular *trans*-Golgi network play essential roles in autophagosome formation to provide a template for lipidation of microtubule-associated protein 1A/1B-light chain 3 and induce further ubiquitination. Golgi-related proteins regulate formation of autophagosomes, and disrupted formation of autophagy can influence Golgi function. Notably, aberrant autophagy has been demonstrated to be implicated in neurological diseases. Thus, targeted therapies aimed at protecting the Golgi or regulating Golgi proteins might prevent or ameliorate autophagy-related neurological diseases. Further studies are needed to investigate the potential application of Golgi therapy in autophagy-based neurological diseases.

## Functions of the Golgi Apparatus

**FIGURE 1:**
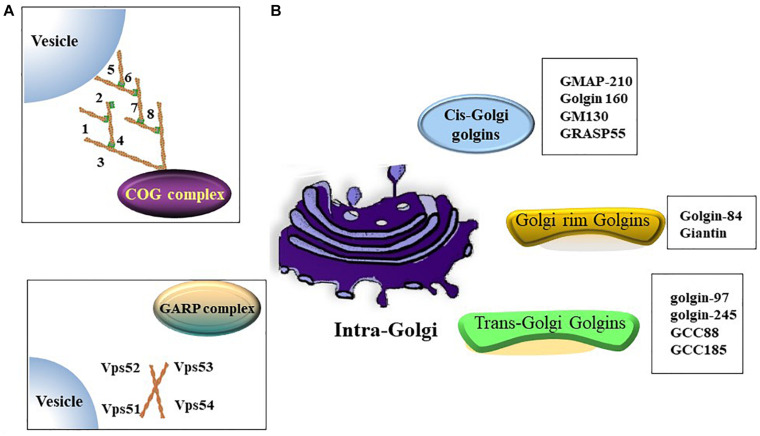
Golgi proteins involved in vesicles transportation. **(A)** Both of oligomeric Golgi (COG) complex (comprised of Cog1-8) and Golgi-associated retrograde protein (GARP) complex (comprised of VPS51, VPS52, VPS53 and VPS54) are tethering complex that functions as cargo trafficking complex. **(B)** A summary of golgins in different zones.

The Golgi apparatus (Golgi hereafter) is a processing and dispatching station, whereby newly synthesized soluble and transmembrane proteins, as well as lipids, are sorted for subsequent transport to the cell surface, secretory granules, or the endosomal system (Rohn et al., 2000; Viotti, 2016). Morphologically, the Golgi comprises associated vesicles and numerous flattened, stacked sacs that are known as cisternae (Rambourg and Clermont, 1990; Koga and Ushiki, 2006). Golgi stacks are collected near the minus ends of microtubules and laterally linked by dynamic fusion events to form a ribbon-like network (Rambourg and Clermont, 1990; Jarvela and Linstedt, 2014). The functional structure of the Golgi includes the *cis*-Golgi, medial Golgi, and *trans*-Golgi networks. In the secretory pathway, endoplasmic reticulum (ER)-Golgi intermediate compartment (ERGIC) is a tubulo-vesicular compartment closely associated with the ER-exit sites (ERES) (Liu et al., 2020) and functions as post-ER sorting stations (Appenzeller-Herzog and Hauri, 2006). Cargos derived from the ER enter a Golgi stack at its *cis*-side and then sequentially pass through medial and *trans*-cisternae (Tie et al., 2017). The *trans*-Golgi network sorts a variety of transport carriers and secretory cargos for delivery to their final destinations at the exit face of the Golgi (Glick and Nakano, 2009; Suda and Nakano, 2012). Golgi proteins involved in vesicle transportation including golgins, conserved oligomeric Golgi (COG) complex and Golgi-associated retrograde protein (GARP) complex etc. (Figure 1).

In addition to the membrane transport and glycosylation functions, the Golgi apparatus is also involved in various biological chemical processes, including mitosis, DNA repair, stress responses, autophagy, apoptosis, and inflammation (Kulkarni-Gosavi et al., 2019). Meanwhile, the biological function of the Golgi is derived from its central position in the secretory pathway and regulatory role in large amounts of signaling molecules (Cancino et al., 2013).

## Formation of Autophagy

Autophagy is a degradation process of the damaged subcellular organelles and improperly folded proteins in the cytoplasm digested by lysosomal lytic enzymes (Arakawa et al., 2017). There are at least three types of autophagic processes in mammalian cells: (i) macro-autophagy coordinates the assembly of autophagy-related (ATG) proteins to generate autophagosomes that are subsequently fused with the lysosomal membrane (Nakatogawa et al., 2009), (ii) chaperone-mediated autophagy delivers KFERQ-like motif-bearing proteins to the lysosomes for degradation via chaperone heat shock cognate protein 70 and cochaperones (Kaushik and Cuervo, 2018), and (iii) micro-autophagy is involved in the bulk degradation of proteins and organelles through invaginations at the lysosomal membrane (Muller et al., 2000; Scrivo et al., 2018). In addition, macro-autophagy includes ATG5/7-dependent canonical and ATG5/7-independent non-canonical autophagy (Nishida et al., 2009). “Macro-autophagy” is referred to as “autophagy” hereafter in this review.

**FIGURE 2:**
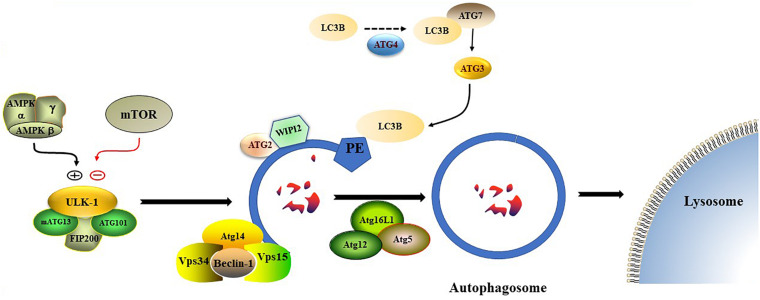
The formation process of autophagosome. Macro-autophagy coordinates the assembly of autophagy-related (ATG) proteins to generate autophagosomes that are subsequently fused with the lysosomal membrane.

Nonselective autophagy may be divided into initiation, elongation/expansion of the isolation membrane, and completion and subsequent fusion of the autophagosome with the lysosome (Zahoor and Farhan, 2018). There are some differences in autophagy-related genes and functions between mammals and yeasts (Li and Zhang, 2019). For instance, ATG29 and ATG31 are yeast specific in the ATG1-complex during the induction phase of autophagy (Li and Zhang, 2019). Functionally, mammalian ATGs can be subdivided into six functional clusters (Wesselborg and Stork, 2015; Table 1). During the initial phase of autophagy, the mammalian autophagy complex- unc-51-like kinase 1 (ULK1) complex is activated and then binds to and phosphorylates ATG9 on the vesicles (Li and Zhang, 2019). Upon the induction of autophagy, ATG9 may first accumulate in tubular vesicular compartments known as ATG9 compartments/reservoir (De Tito et al., 2020). After induction of autophagy, Beclin-1 and its multiple modifiers (Kihara et al., 2001) associated with the ATG system fulfill the elongation of isolation membranes/phagophores (Kihara et al., 2001; Mizushima et al., 2011). Finally, ATG8 (including microtubule-associated protein 1A/1B-light chain [LC]3b and GABARAPS) recognizes ubiquitinated targets. P62 binds to ubiquitin and ATG8 proteins via its C-terminal UBA domain (Zaffagnini et al., 2018) and delivers ubiquitinated cargos for autophagic degradation (Johansen and Lamark, 2011; Liu et al., 2016; Figure 2).

**TABLE 1:** Function of ATGs in different steps during autophagosome biogenesis.

**Autophagy Process**	**Mammals**	**Yeast**
Initiation of autophagosome	ULK1–ATG13–FIP200–ATG101 protein kinase complex	ATG1-ATG13-ATG17-ATG11-ATG29-ATG31 complex
Nucleation of phagophores	PtdIns3K class III complex (VPS34, the adaptor VPS15 (p150), and Beclin-1 (ATG6), ATG14L, NRBF2)	PtdIns3K class III complex (ATG34, ATG15, ATG6, ATG14/ATG38)
Formation of phagophores (membrane source)	Multi-spanning transmembrane protein ATG9A	Multi-spanning transmembrane protein ATG9A
Expansion of autophagosomes	PtdIns3P-binding WIPI/ATG18–ATG2 complex	PtdIns3P complex (ATG18, ATG21 and ATG2)
Expansion of autophagosomes	Ubiquitin-like ATG5/ATG12 system	Ubiquitin-like ATG5/ATG12 system
Expansion of autophagosomes	Ubiquitin-like ATG8/LC3 conjugation system	Ubiquitin-like ATG8/LC3 conjugation system

*Ulk1, unc-51 like autophagy activating kinase 1.*

Autophagy has a variety of signal regulatory pathways. Starvation-induced autophagy is regulated by inactivation of the mammalian target of rapamycin (mTOR) and activation of AMP kinase (AMPK) (Rubinsztein et al., 2012). In addition, ULK1 is an effector of the two kinases (Egan et al., 2011; Kim et al., 2011). Another regulatory pathway of autophagy is the cAMP–Epac–PLCε–IP3-calcium pathway (Metcalf et al., 2012).

## Interaction Between the Golgi Apparatus and Autophagy

In mammalian cells, the Golgi is a cell sensor because various signaling factors are located in or transduced to the apparatus (Cancino et al., 2013). Autophagy could be influenced by dysfunction of the Golgi. As a signaling platform, the Golgi provides not only a membrane for autophagosome formation but also a location for induction and elongation of autophagosomes. Moreover, Golgi-related proteins participate in autophagosome formation directly. Conversely, moderate autophagy can help maintain the normal function of the Golgi apparatus.

### Golgi Influences the Signaling Pathway of Autophagy

#### The Golgi Is Involved in Autophagosome Formation by Regulating Transport of ATG9

**FIGURE 3:**
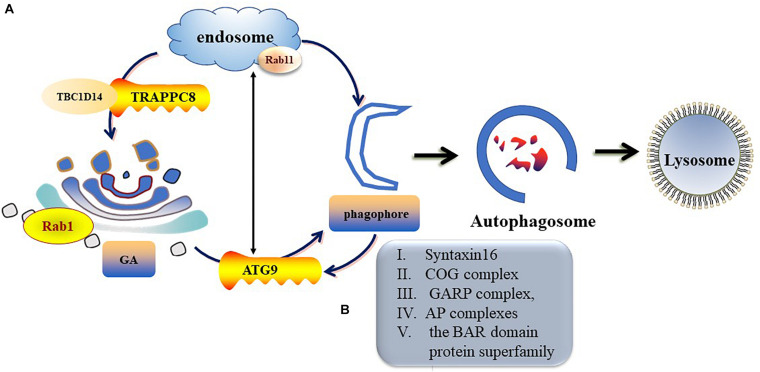
Trafficking of mATG9 in different conditions. **(A)** Under nutrient-rich conditions, ATG9 is mainly colocalizes with TGN and endosomes. During starvation-induced autophagy, mAtg9 redistributes from TGN to juxtanuclear region. ATG9 relocate to the Golgi from RAB11-positive recycling endosomes to RAB1-positive Golgi compartments is controlled by a complex including TBC1D14 and mammalian TRAPPIII. **(B)** Syntaxin16, oligomeric Golgi (COG) complex, Golgi-associated retrograde protein (GARP) complex, adaptor protein (AP) complexes and the BAR domain protein superfamily take part in the regulation of ATG9 trafficking.

ATG9 is the sole multi-spanning transmembrane protein among core ATG proteins and is essential for autophagy (Kakuta et al., 2012; Imai et al., 2016). The mechanisms regulating ATG9 trafficking may differ between the species (Webber and Tooze, 2010). In mammalian cells, ATG9-containing vesicles cycle between the *Trans*-Golgi Network (TGN), post-Golgi, and endosomal compartments (Mari et al., 2010; Imai et al., 2016) and form new autophagosomes in response to stresses (Zhou et al., 2017). Under nutrient-rich conditions, ATG9 is mainly co-localizes with TGN markers of the Golgi and recycling endosomes (Orsi et al., 2012). During starvation-induced autophagy, membrane ATG9 (mATG9) localizes to both a juxtanuclear region, which corresponds to the TGN, and a peripheral population, which partially co-localizes with late endosomes (Zhou et al., 2017). Initiation and formation of the autophagosome in mammalian cells are through the formation of the omegasome (Mizushima et al., 2011; Yu et al., 2018). Retrieval of ATG9 to the Golgi from RAB11-positive recycling endosomes to RAB1-positive Golgi compartments is controlled by a complex including TBC1D14 and mammalian transport protein particle (TRAPP) III (Lamb and Tooze, 2016; Lamb et al., 2016). TRAPPC8 forms part of a mammalian TRAPPIII-like complex required for RAB1 activity and directed to the omegasome (Lamb and Tooze, 2016). In addition, TRAPPC8 is needed for maintenance of Golgi integrity (Lamb et al., 2016). Thus, abnormal function or morphology of the Golgi may influence the transportation of ATG9 from the Golgi to the destined organelle and ultimately disrupt autophagosome formation (Figure 3).

#### Golgi Fragmentation Is Accompanied by Autophagy Induction

α Soluble N-ethylmaleimide sensitive factor attachment protein alpha (αSNAP) is a well-known component of the vesicle trafficking machinery (Naydenov et al., 2012a, 2014) and mediates vesicle transport from the ER to the *cis*-Golgi (Peter et al., 1998). Loss of αSNAP impairs Golgi-dependent glycosylation (Naydenov et al., 2014), triggers Golgi fragmentation, and induces autophagy (Naydenov et al., 2012b). By controlling the integrity of the Golgi, αSNAP inhibits mTOR-related signaling, limits the membrane supply for autophagosomal biogenesis, and negatively regulates autophagy (Naydenov et al., 2012b). Additionally, depletion of another multi-subunit tether factor, Golgi-associated retrograde protein (GARP), also causes autophagy defects (Perez-Victoria et al., 2010). The GARP complex is a multi-subunit complex made of four distinct proteins (VPS51, VPS52, VPS53, and VPS54) localized in the TGN and involved in endosome-to-TGN retrograde transport (Hirata et al., 2015; Uwineza et al., 2019). Dysfunction of the GARP complex may influence transport of the retrograde vesicle and sorting of proteins in the Golgi apparatus (Schmitt-John, 2015). GARP complex is the effector that connects Arl1 and Ypt6 with autophagy (Yang and Rosenwald, 2016), and it may cause autophagy defects in mATG9 that is not transported or is unable to obtain sufficient membrane support because of disrupted integrity of the Golgi (Eapen and Haber, 2013). Moreover, conserved oligomeric Golgi (COG) complex mutants exhibit defective autophagy (Wang I. H. et al., 2017). The COG complex, which consists of eight subunits (COG 1-8), is a multi-subunit vesicle tethering complex that functions in retrograde trafficking at the Golgi (D’Souza et al., 2019). Similar to the depletion effect of GARP mentioned above, defective autophagy that is caused by COG complex malfunction is also associated with blocked transportation of ATG9 and insufficient membrane support derived from abnormal function of the Golgi (Ohashi and Munro, 2010). Moreover, the COG complex localizes to the pre-autophagosomal structure and interacts with ATG12, ATG17, ATG20, and ATG24 (Yen et al., 2010). Thus, autophagy is dependent on normal function and integrity of the Golgi because the loss of Golgi-related proteins, including αSNAP, GARP, or COG, can result in aberrant autophagy.

#### ERGIC and TGN Provide a Location for Formation of Autophagosomes

The ERGIC functions as the membrane provider that triggers LC3 lipidation and is required for autophagosomal biogenesis (Ge et al., 2013). LC3 is linked to phosphatidylethanolamine on autophagosomal precursor membranes for lipidation which is mediated by the E3-like Atg12-Atg5⋅Atg16 complex (Ge et al., 2015). The above process is modulated by the phosphatidylinositol 3-kinase (PtdIns3K) complex, which consists of ATG14, Beclin-1, PIK3C3/VPS34, and PIK3R4/VPS15/p150. Beclin-1-PtdIns3K complexes are concentrated in the TGN (Shoji-Kawata et al., 2013) and play a role in the sorting of autophagosomal components and lysosomal proteins (Kihara et al., 2001). The ERGIC may play a role in an early stage of phagophore formation by providing a platform to recruit the class III PI3K complex and provide precursor membranes for phagophore initiation (Ge and Schekman, 2014; Ge et al., 2014). Additionally, ATG14 directs Beclin-1/Atg6 from the TGN to the autophagosomes (Sun et al., 2008). Therefore, ERGIC and TGN provide a location for the induction and elongation of autophagosomes.

### Golgi-Related Proteins Are Directly Involved in Formation or Regulation of Autophagy

**FIGURE 4:**
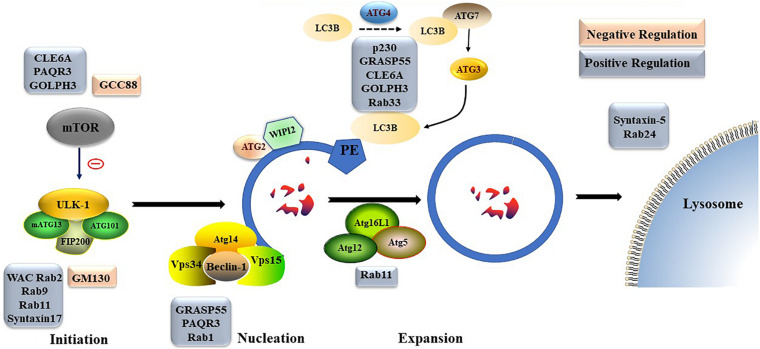
The detailed regulation mechanisms of Golgi proteins involved in autophagosome formation.

Different Golgi proteins play different roles in formation and degradation of autophagosomes. For example, Golgi coiled-coil protein (GCC)88 and Golgin subfamily A member 2 (GM130) play negative regulatory roles in formation and degradation of autophagosomes, while p230, WW domain containing adaptor with coiled-coil (WAC), Rabs, Golgi reassembly-stacking protein of 55 kDa (GRASP55), CLE6A, progestin and AdipoQ Receptor 3 (PAQR3), and Golgi phosphoprotein 3 (GOLPH3) play positive regulatory roles in formation and degradation of autophagosomes. The detailed underlying mechanisms, including transportation of mATG9; regulation of mTOR, ULK, homotypic fusion and protein sorting complex, or PtdIns3K UV radiation resistance-associated gene protein (UVRAG) complex; and late steps of macroautophagic and endocytic degradation, are described below (Figure 4).

#### Golgin-245 (p230)

Golgin-245 or t-golgin-1 is a member of peripheral membrane proteins that associate with the TGN via a C-terminal GRIP domain (Yoshino et al., 2005), and dimerization of the GRIP domain is essential for its Golgi localization (Lu et al., 2006). P230 participates in cargo trafficking and regulates the position of the Golgi indirectly probably by regulating the retrograde movement of cargos or activation of dynein-dynactin complexes in newly formed Golgi elements (Yoshino et al., 2005). p230 poses a positive regulation in autophagy. During amino acid starvation, p230 is essential to the formation of LC3-positive structures in autophagosome (Sohda et al., 2015). The potential mechanism derived that p230 provides a molecular link of specific vesicles with mAtg9 from the TGN to the target structures (Sohda et al., 2015).

#### GCC88

GCC88 negatively regulates the Golgi ribbon, which involves the alteration in actin cytoskeleton. Studies have reported that knock-down of GCC88 results in a longer Golgi ribbon (Gosavi et al., 2018). Scaffold intersectin-1 (ITSN-1) is a scaffolding protein involved in endocytosis and has guanine nucleotide exchange activity for Cdc42 (Zamanian and Kelly, 2003), and it is a novel TGN component and a binding partner of GCC88 that links the Golgi complex to the actin cytoskeleton to regulate the Golgi structure (Makhoul et al., 2019). The GCC88-ITSN-1 pathway is relevant to several different processes that lead to abnormal morphology of the Golgi (Makhoul et al., 2019). Alteration in Golgi architecture does not affect membrane transport, while organization of the Golgi ribbon has a regulation effect on autophagy via mTOR signaling (Gosavi et al., 2018). mTOR is one of the main regulators of autophagy by controlling activity of the ULK1 complex (Al-Bari and Xu, 2020). Loss of the Golgi ribbon results in dramatic reduction in mTOR activity and induction of autophagy, presumably due to the inability to recruit mTOR to the Golgi pool (Gosavi et al., 2018).

#### GM130 and WAC

GM130 is located in the *cis*-Golgi, and knockout of GM130 causes Golgi fragmentation and impaired secretory trafficking in mice (Liu et al., 2017). WAC localizes in the nucleus and the Golgi, where it regulates epigenetics and post-mitotic Golgi reassembly, respectively (Joachim and Tooze, 2016). Two Golgi proteins, WAC and GM130, are interacted with the ATG8 homolog (GABARAP) and influence the autophagy signaling pathway by regulating the subcellular localization of GABARAP (Joachim et al., 2015). WAC promotes autophagy, while GM130 inhibits autophagy (Joachim et al., 2015). The GABARAP subfamily is critical to starvation-induced activation of autophagy and specifically promotes ULK kinase activation dependent on the ULK1 LIR motif (Slobodkin and Elazar, 2013). GABARAP located in the Golgi can inhibit autophagy, while the pericentriolar matrix (PCM) pool of GABARAP facilitates formation of autophagosome (Joachim et al., 2015; Joachim and Tooze, 2016). In steady-state conditions, GABARAP is bound to GM130 on the Golgi complex (Joachim et al., 2015). Amino acid starvation promotes activation of WAC by promoting its association with GM130 and dissociation of GABARAP from GM130 (Slobodkin and Elazar, 2013; Joachim and Tooze, 2018). Upon release from the Golgi complex, GABARAP accumulates in the PCM, and the reservoir of GABARAP in PCM activates the ULK complex and induces autophagy (Joachim and Tooze, 2018).

#### Rabs (Small GTP Binding Proteins)

Rab protein is the largest subfamily of the small molecule GTP-binding protein family, consisting of the conserved G domain and highly variable N-terminal and C-terminal. Rab proteins not only participate in maintaining integrity of the Golgi but also play a regulatory role in signal pathways or cargo trafficking in the Golgi. In addition, Rab GTPases that are critical to the function of the Golgi have been demonstrated to take part in induction, elongation, and degradation of autophagosomes, as shown in Table 2. Rab proteins, many with ATG proteins as interacting partners, modulate ULK1 kinase activity, recruit the ATG5–12/16L complex, and tether ATG9 vesicles or regulate endosomal degradation, which composes the necessary steps in the early or late stage of autophagy (Morgan et al., 2019).

**TABLE 2:** The function of Rabs in autophagy.

**RABS**	**Golgi Localization**	**Function in Golgi**	**Role in Autophagy**
Rab1	Golgi stack, ERGIC	Mediate dynamic membrane trafficking between ER and Golgi (Liu and Storrie, 2012; Yang et al., 2016)	Regulates macro-autophagy by recruiting its effector, Atg1, to the PAS to tether Atg9 vesicles to each other or to other membranes (Wang et al., 2013) Supply of membranes from source organelles or extension of the IM (Kakuta et al., 2017) Recruit and activate ULK1 and regulate PI (3)P biogenesis (Feng et al., 2018; Toh et al., 2020)
Rab2	Co-localization with GOLGA2/GM130, ERGIC	Regulate ER-to-Golgi transportation and Golgi organization (Liu and Storrie, 2012)	Modulate ULK1 kinase activity to propagate signals for autophagosome formation (Ding et al., 2019) Interact with autophagosomal RUBCNL/PACER and STX17 to further specify the recruitment of HOPS complex for autolysosome formation (Ding et al., 2019)
Rab9a	TGN	Transport of protein from late endosomes to the TGN (Dhingra et al., 2019)	Co-localized with autophagosomes and autolysosomes and associated with Ulk1 pathway involved in mitophagy—a specialized form of autophagy (Dhingra et al., 2019) Rab9 mediate extension and closure of isolation membranes in the alternative autophagy pathway replaces Atg5- and Atg7-dependent autophagy (Arakawa et al., 2017; Shimizu, 2018)
Rab11	TGN	Mediates proteins sorting for anterograde TGN-plasma membrane transport (Parmar and Duncan, 2016)	Promote constitutive ATG9 cycling from the Atg9 compartment through its recruitment of TRAPP and activation of RAB1 (Lamb et al., 2016) RAB11A-positive membranes act as a platform on which autophagosomes assemble by favoring the recruitment of the Atg16L complex (Li et al., 2017; Puri et al., 2018) Rab11 mediates vesicle formation from the recycling endosome directed to forming autophagosomes during amino acid starvation (Longatti et al., 2012)
Rab18	*cis*-Golgi	ER/Golgi trafficking and Golgi organization (Martin et al., 2005)	The stable loss of RAB18 results in a reduced LD-derived lipid availability that provokes adaptive alterations of the autophagy network to maintain autophagic activity (BasuRay, 2019; Bekbulat et al., 2020)
Rab24	*cis*-Golgi and ERGIC	Undefined	Rab24 localizes to autophagosomes, and that its distribution changes remarkably upon shifting to deprivation of nitrogen nutrients, a physiological stimulus for autophagy (Munafo and Colombo, 2002) RAB24 functions in membrane fusion events during the late steps of macroautophagic and endocytic pathways (Yla-Anttila and Eskelinen, 2018) Rab24 co-localized with LC3 and interacts with Rab7 to regulate endosomal degradation (Munafo and Colombo, 2002; Chua et al., 2011)
Rab41	TGN	Anterograde trafficking of cargo from the ER to the Golgi and the maintenance of Golgi integrity (Liu et al., 2013)	Cargo–receptor recruitment at the early stages of autophagic recognition (Ogawa et al., 2018)
Rab33	Medial Golgi cisternae	The structural Organization of the Golgi apparatus, and retrograde Golgi-to-ER transport (Chua et al., 2011)	Regulation by its GAP protein (OATL1) is necessary to ensure autophagosome maturation (Fukuda and Itoh, 2008; Itoh et al., 2008) Rab33 regulates autophagosome formation through promoting LC3-PE conjugation to recruit the Atg5–12/16L complex (Fukuda and Itoh, 2008; Chandra et al., 2016).

*HOPS complex, Homotypic fusion and Protein Sorting Complex; IM: isolation membrane; RUBCNL/PACER: rubicon like autophagy enhancer; STX17: syntaxin 17; Ulk1: unc-51 like autophagy activating kinase 1; PI (3)P: phosphatidylinositol 3-phosphate; PAS: pre-autophagosomal structure; TRAPP: transport protein particle.*

#### GORASP2/GRASP55

GRASP55 is primarily localized on the medial-*trans* cisternae, plays a role in medial-*trans* Golgi stack formation to maintain the number of cisternae per stack, and links the Golgi ribbon (Xiang and Wang, 2010). Depletion of GRASP55 impairs accurate protein glycosylation and sorting (Zhang and Wang, 2015). Moreover, GRASP55 also functions as an energy sensor and membrane linker in autophagy (Zhang et al., 2018). Upon glucose starvation, GORASP2 is de-O-GlcNAcylated and acts as a membrane tether to facilitate autophagosome-lysosome fusion (Zhang et al., 2018). Additionally, GORASP55 interacts with BECN1 to facilitate the assembly and membrane association of the PtdIns3K UVRAG complex (Zhang et al., 2019). Furthermore, GRASP55 displays positive regulatory effects on autophagy induction and influences the lipidation of LC3b (Dupont et al., 2011).

#### CLEC16A

CLEC16A is important for the morphology maintenance of the Golgi in Purkinje cells, which may be derived from its function in maintaining or assisting other Golgi apparatus proteins in organelle structures (Redmann et al., 2016). Golgi-associated CLEC16A co-localizes with active mTOR complex and may function as a negative regulator in starvation-induced autophagy via enhancing the mTORC1 signaling pathway (Tam et al., 2017). Moreover, CLEC16A deficiency results in a failure of autolysosome function or clearance in the step-in autophagy downstream of autophagosome and lysosome fusion (Redmann et al., 2016).

#### PAQR3

PAQR3 is a 7-transmembrane protein specifically localized in the Golgi apparatus (Li et al., 2018). PAQR3 can augment autophagy via different mechanisms. PAQR3 can not only enhance the capacity of pro-autophagy class III PI3K but also integrate AMPK signal to activation of ATG14L-linked VPS34 complex upon glucose starvation (Xu et al., 2016). Besides the initiation role in the early stage of autophagy induced by glucose starvation, PAQR3 also negatively regulates amino acid-induced activation of mTORC1 (Wang L. et al., 2017). In addition, Cao et al. demonstrated that PAQR3 modulates tyrosine kinase inhibitor-induced autophagy in lung cancer cells by blocking epidermal growth factor receptor interaction with BECN1 and inhibiting BECN1 phosphorylation (Cao et al., 2020).

#### GOLPH3

GOLPH3, which is also known as MIDAS, GOPP1, or GPP34, is a peripheral membrane protein localized in the Golgi (Yu et al., 2016; Kuna and Field, 2019). GOLPH3 localized to the *trans*-Golgi provides a link from the *trans*-Golgi membrane to the actin cytoskeleton that plays a critical role in anterograde trafficking to the plasma membrane (Buschman et al., 2015). GOLPH3 affects autophagy through different mechanisms. For instance, Li et al. showed that during the initial oxygen-glucose deprivation injury, suppression of GOLPH3 led to a significant reduction in reactive oxygen species (ROS) generation and directly affected stress-related autophagy by suppressing lipidation of LC3b (Li et al., 2016). Moreover, GOLPH3 overexpression activates autophagy inhibitor mTOR-signaling and confers autophagy resistance (Scott et al., 2009; Salem et al., 2012).

#### Protein-Mediated Trafficking at the Golgi

The Golgi provides a locale for the formation of a variety of cargo-containing vesicles targeted to their destinations by binding with different protein coats (De Tito et al., 2020). Among various coats involved in TGN exit, adaptor protein (AP) complexes and the BAR domain protein superfamily, including bax-interacting factor 1 (Bif-1)/SH3GLB1, Arfaptins, and PI4KIII, are implicated in the regulation of ATG9A trafficking and are firmly connected with autophagy (De Tito et al., 2020). AP-1, 2, and 4, as sorting motifs, bind with ATG9 during trafficking from recycling endosomes to the Golgi (Imai et al., 2016). Bif-1-mediated fragmentation of the Golgi complex during nutrient starvation plays a crucial role in ATG9 trafficking and autophagosome biogenesis through the Bif-1-dynamin 2 interaction (Takahashi et al., 2011). Arfaptin2 regulates the starvation-dependent distribution of ATG9A vesicles, and PI4KIIIβ interacts with ATG9A and ATG13 to control PI4P production in the autophagic response (Imai et al., 2016).

#### Soluble N-Ethylmaleimide-Sensitive-Factor-Attachment Protein Receptors (SNAREs)

Accumulating evidence demonstrates that Syntaxin (Stx)5, Stx17, and Stx16 are involved in the autophagy pathway (Tang, 2019). Stx17 is a SNARE protein mainly localized in the ER and partly in the ERGIC (Muppirala et al., 2011) and is essential for maintaining the architecture of ERGIC and the Golgi (Muppirala et al., 2011), and it contributes to the autophagy pathway at several stages from initiation to maturation (Gu et al., 2019). Kumar et al. revealed that TBK1-phosphorylated Stx17 is important for assembly of the ULK1 complex and is critical for autophagy initiation (Kumar et al., 2019). In addition, Stx17 interacts with mAtg8s via the LIR motif and inserts into the autophagosomal membrane (Kumar et al., 2018; Shen et al., 2020). Stx16 is localized in the late Golgi compartments, receives retrograde transport from the endosomes (Xu et al., 2002), and is involved in autophagy by facilitating the transport of ATG9a-containing vesicles to growing autophagosomes (Tang, 2019). Moreover, stx16 is recruited by Atg8/LC3/GABARAP family proteins to autophagosomes and endolysosomes (Tang, 2019). Stx5 is involved in ER-Golgi trafficking of specialized cargo molecules, and its proper expression maintains the normal morphology and function of the Golgi in mammalian cells (Linders et al., 2019). The STX5 partner SNARE GS27 binds to both COG6 and COG8, and the COG complex plays a regulatory role in the *cis*-Golgi-localized STX5 SNARE complex (Willett et al., 2013). Depletion of syntaxin-5 complex components results in accumulation of autophagosomes due to lysosomal dysfunction, thereby leading to decreased degradation of autophagic substrates (Renna et al., 2011).

### Effects of Disrupted Formation of Autophagy on Golgi Function

#### Autophagy May Induce the Golgi Stress Response by Activating TFE3

The Golgi stress response, a mechanism to meet cellular demands, maintains the homeostasis of the Golgi under the conditions of the overloading state (Taniguchi et al., 2015). Sensors of Golgi stress detect the accumulation of proteins that are not properly modified (Taniguchi and Yoshida, 2017). Then, sensors activate the following three downstream signaling pathways related to the Golgi response to stress: TFE3 (inducing Golgi expansion by regulating the transcriptional induction of Golgi-related genes), HSP47 (preventing Golgi stress-induced apoptosis), and CREB3 (inducing apoptosis through the transcriptional induction of ARF4) (Taniguchi and Yoshida, 2017). One previous study found that autophagy initiation allowed for the parallel activation of the TFE3 pathway (Mytych et al., 2020). Additionally, it has been proposed that core proteins accumulate in the Golgi, cause Golgi dysfunction, and induce autophagy of the Golgi apparatus. Subsequently, signal-inducing autophagy indirectly activates TFE3 (Taniguchi et al., 2015; Mytych et al., 2020). After activating TFE3, the Golgi expands in accordance with greater protein synthesis demands.

#### Non-Canonical Golgi Membrane-Associated Degradation (GOMED) Promotes Alternative Clearance in Autophagy-Deficient Cells

The molecular mechanisms of macro-autophagy involve more than 30 autophagy-related proteins (ATGs) (Mizushima et al., 2011), and ATG5 and ATG7 are key components in the formation of autophagosomes and are essential for macro-autophagy (Su et al., 2017). Nishida et al. (2009) identified two distinct autophagic pathways: the conventional autophagy pathway and the alternative autophagy pathway, the latter one was demonstrated as an ATG5/ATG7-independent type of autophagy and named “alternative autophagy.” Different from the conventional autophagy, the autophagic membrane source of “alternative autophagy” is exclusively derived from Golgi apparatus (Arakawa et al., 2017). A novel degradation pathway, named Golgi membrane-associated degradation (GOMED) pathway, is activated when Golgi-to-plasma membrane trafficking is disrupted due to lack of Atg5/Atg7-dependent autophagy (Yamaguchi et al., 2016). When this pathway is activated, the Golgi membranes become stacked, elongated, and curved and generate double-membrane compartments that enclose the cytoplasm and various organelles (Yamaguchi et al., 2016); this process is also classified as “alternative autophagy.” Furthermore, autophagy inhibition can trigger a compensatory clearance by GOMED when autophagic lysosomal degradation is impaired (Barthet and Ryan, 2018).

## Aberrant Autophagy is Involved in Neurological Diseases

Autophagy is involved in tumor suppression, resistance to pathogens, and extension of lifespan (Dupont et al., 2011). It is worth noting that moderate levels of autophagy are important for the proper functioning of the nervous system. Autophagy plays physiological roles in neurodevelopment and maintenance of neuronal homeostasis, and it is involved in pathological processes that affect the brain, including neurodegenerative disorders and stroke (Corti et al., 2020).

### Stroke

Ischemic stroke produces ROS and induces oxidative stress. In response to stress, autophagy exerts a two-way effect in ischemic stroke, and cerebral ischemic injury can be reduced by regulating autophagy (Mo et al., 2020). By enhancing autophagosome formation and promoting fusion of autophagosomes or attenuating autophagic-lysosomal defects, the autophagy-lysosome pathway exerts a protective effect on cerebral ischemia; however, promoting lysosomal dysfunction and autophagic defect exerts a detrimental effect (Chen et al., 2020). The major regulatory pathways of autophagy in cerebral ischemic injury involve the PI3K/Akt/mTOR pathway (inhibit), PI3K pathway (type I PI3K, inhibit; type III PI3K, enhance), the PPAR-γ/Beclin-1 pathway (enhance), the MAPK pathway (enhance), the NF-κB-dependent p53 signaling pathway (enhance) (Wolf et al., 2019), the HIF-1α/AMPK/mTOR signaling pathway (enhance), and the HIF-1α/BNIP3 signaling pathway (enhance) (Sun et al., 2018).

### Neurodegenerative Disorders

#### Alzheimer’s Disease (AD)

Alzheimer’s disease is characterized by progressive dementia and cognitive impairment (Djajadikerta et al., 2020). Its pathogenesis is presented as intracellular accumulation of hyperphosphorylated tau protein and extracellular aggregates of amyloid β (Aβ) (Djajadikerta et al., 2020; Tiwari et al., 2019). Autophagy plays a dual role in Aβ degradation and secretion. In the early stages of AD, Aβ formation can activate autophagy, and Aβ can be degraded via transportation from autophagosomes to lysosomes. In the later stages of AD, the persistent accumulation of Aβ induces aberrant autophagy and excessive phagocytosis of normal proteins (Kuang et al., 2020). Further, Aβ-induced autophagy impairment that is mediated by the loss of Beclin-1 leads to microglial activation and increased production of proinflammatory cytokines that contribute to neuroinflammation (Thangaraj et al., 2020). Moreover, Aβ accumulation leads to decreased Bif-1 expression in neurons, which in turn elevates Aβ accumulation and plaque formation, possibly through impairment of autophagy (Wang et al., 2015).

#### Parkinson’s Disease (PD)

Parkinson’s disease is a progressive neurological disease characterized by Lewy bodies, which are composed of aggregates of α-synuclein (Dickson, 2018). α-synuclein is considered the typical pathologic correlate of PD (Dickson, 2018) and is composed of an N-terminal of amphipathic α-helical domain combining with the membrane (Deng et al., 2018). It is encoded by the synuclein alpha (SNCA) gene, and most SNCA duplication carriers are associated with parkinsonism and nonmotor symptoms (Chartier-Harlin et al., 2004; Deng et al., 2018). Mutations in α-synuclein, such as the mutant SNCA A53T, result in significantly decreased lipidation of LC3 and increased activity of mTOR signaling pathway, which inhibit the autophagic flux and reduce removal of abnormal proteins (Jiang et al., 2013). Besides mutations in SNCA, other genetic forms of PD involve mutations in LRRK2, VPS35, PRKN, PINK1, and PARK7 (Hou et al., 2020). Different PD genes/mutations affect autophagic function and contribution of autophagic impairment to PD pathogenesis (Hou et al., 2020).

#### Huntington’s Disease (HD)

Huntington’s disease is characterized by a pathologic mutation consisting of an expanded CAG repeat in the huntingtin gene (HTT) on chromosome 4, encoding the huntingtin (htt) protein (Dayalu and Albin, 2015). Htt functions as a scaffold protein that is required for selective autophagy (Ochaba et al., 2014; Rui et al., 2015). Htt positively modulates selective autophagy by facilitating p62-mediated cargo recognition or promoting ULK1 activation (Rui et al., 2015). Mutant HTT (mHTT) could affect mitophagy by interfering with ULK1 activation and shift from the mTORC1 to HTT scaffolding complexes and impairing the interaction of BECN1-PIK3R4/VPS15 (Franco-Iborra et al., 2020). Impaired autophagy leads to impaired clearance of abnormal proteins.

#### Amyotrophic Lateral Sclerosis (ALS) and Frontotemporal Dementia (FTD)

Amyotrophic lateral sclerosis and FTD are mainly caused by genetic mutations that lead to abnormal protein aggregates, such as RNA-binding protein and TAR DNA-binding protein-43 kDa, in the brain (Hedl et al., 2019). More than 25 genes that are implicated in ALS, FTD, and ALS/FTD have been identified (Ling et al., 2013). There are a multitude of ALS genes with functions related to the autophagic system, including p62, optineurin, VCP, ubiquilin 2, and TBK1 (Nguyen et al., 2019). Among the most frequently identified mutations in ALS, mutations in superoxide dismutase 1 (SOD1) can influence autophagy (Casterton et al., 2020). Mutant SOD1 fails to interact with Beclin-1 and destabilizes Beclin-1-Bcl-xL complex or inhibits mTOR signaling, leading to a defective autophagy flux (Lee et al., 2015; Nguyen et al., 2019). Additionally, mutations in microtubule-associated protein tau or granulin (GRN) are associated with pure FTD. GRN is a gene that encodes progranulin (PGRN) (Holler et al., 2017). PGRN can regulate lysosome function, and PGRN deficiency in neurons increases autophagy and causes abnormally enlarged lysosomes (Elia et al., 2019).

### Traumatic Brain Injury (TBI)

Traumatic brain injury is defined as a traumatically induced structural injury or physiological disruption of brain function as a result of external forces (Zeng et al., 2020). Neuron dysfunction induced by TBI is derived from primary and secondary brain injury mechanisms, and secondary injury results from delayed neurochemical, metabolic, and cellular changes (Feng et al., 2017). Further, disruption of autophagy flux is one of the mechanical attributes to secondary injury (Feng et al., 2017; Wu and Lipinski, 2019). Autophagy has a double regulating effect in the pathological process in TBI. Results from a previous study revealed that LC3-II, Beclin-1, p62, and autophagosomes were increased in human TBI autopsy samples (Sakai et al., 2014), suggesting enhancement of autophagy initiation. Conversely, TBI activates phospholipase A2 and attenuates lysosomal membrane permeability, as well as subsequent impairment of autophagy and neuronal loss (Sarkar et al., 2020).

### Subarachnoid Hemorrhage (SAH)

Subarachnoid hemorrhage is a subtype of hemorrhagic stroke with high mortality and morbidity that are mainly caused by early brain injury (EBI) (Liu et al., 2014). Elevated autophagy levels and activated autophagy-related pathway proteins, which exert a protective effect, have been demonstrated in experimental SAH models (Wang et al., 2012). Li Y. et al. (2019) found that rapamycin inhibited mTOR activity and induced autophagy, which subsequently attenuated neuronal injury in EBI with improved neurological deficits, BBB permeability, and brain edema. Li and Han (2018) demonstrated that resveratrol (RSV) attenuated EBI after SAH, and the effects of RSV on SAH-induced EBI were mediated via the AMPK/SIRT1/autophagy pathway.

## Regulation of Golgi Function May Be a Therapeutic Target for Aberrant Autophagy-Based Neurological Diseases

Proper regulation of autophagy can reduce neuronal damage and diminish the disease progression. As we mentioned before, the Golgi plays an important role in regulating autophagy. Therefore, therapies that affect Golgi morphology or function could be used as a therapeutic tool in aberrant autophagy-related diseases. Studies have reported that Golgi therapy alleviates the damage in cancers (Chang et al., 2012; Cao et al., 2019) and autoimmune diseases (Schuster et al., 2015) by influencing the formation of autophagosomes. Since the same autophagy mechanisms are observed in cancers, autoimmune diseases, and neurological diseases, regulation of the function of the Golgi apparatus may be a potential therapeutic target for neurology diseases, and neuron damage in neurological diseases could be alleviated by repairing the Golgi function or regulating Golgi proteins to keep moderate autophagy. In fact, a normal and appropriate level of autophagy plays a role in the maintenance of Golgi homeostasis. For example, (Li D. et al., 2019) found that the upregulation of miR-21-5p inhibited formation of autophagosomes in neurons by targeting Rab11a and subsequently attenuated autophagy-induced nerve injury *in vitro*, and (Li et al., 2016) demonstrated that suppression of GOLPH3 alleviated oxidative injury and directly affected oxidative stress-related autophagy by suppressing lipidation of LC3B in oxygen-glucose deprivation/reoxygenation injury. These findings support the hypothesis that the Golgi may be an ideal target for regulating autophagy disruption in neurodegenerative diseases or stroke. Thus, targeted therapies that are aimed at protecting the Golgi or regulating Golgi proteins may be effective therapeutic approaches that prevent autophagy-related neurological diseases in the future.

## Conclusion

The Golgi plays an important role in autophagy from the early stages of formation to the final degradation. Autophagy-related proteins, including Beclin-1 and ATG9, are localized in the Golgi, and some regulators in autophagy signaling pathways, such as PtdIns3K class III complex and Rabs, are trafficking in ERGIC and TGN. In addition, Golgi-localized proteins, including GRASP55, Golgin230, GM130, and GOLPH3, maintain the function and integrity of the Golgi and regulate the transport and formation of autophagosomes. Moreover, aberrant autophagy is involved in neurological diseases. Therefore, Golgi-targeted therapies that overexpress or silence Golgi proteins involved in autophagy or that interfere with the transport or trafficking functions of the Golgi may be used to treat neurological diseases. Future studies should investigate the potential application of Golgi therapy in autophagy-based neurological diseases.

## Author Contributions

SD carried out the literature review and drafted the manuscript. JL and XW helped draft the manuscript. WL contributed to and finalized the draft. All authors read and approved the final manuscript.

## Conflict of Interest

The authors declare that the research was conducted in the absence of any commercial or financial relationships that could be construed as a potential conflict of interest.
